# Enlargements of the Liver.—V

**Published:** 1910-01-29

**Authors:** 


### ENLARGEMENTS OF THE LIVER.—V.

### CIRRHOSIS—(*Continued*).

Unilobular, or biliary, or hypertrophic cirrhosis is characterised by jaundice, ascites being a most unusual complication. Both this and the ascitic type of cirrhosis already described are most frequently caused by abuse of alcohol. This variety is much rarer than the multilobular type, and it is probably an earlier stage of the disease, as has been mentioned above. The liver is considerably and uniformly enlarged, attaining a much greater size than it does in the multilobular form. It may weigh from 5 to 10 lbs., or more, whereas in the multilobular stage it rarely reaches a weight of 6 lbs., notwithstanding its increase in specific gravity. The surface is quite smooth and the capsule but little thickened. The normal shape of the organ is usually maintained. The edge is sharp, regular, firm, and well defined. On section the cut surface is smooth, and it is often of either a yellowish or a deep green colour. It is tough, but not so hard as is the multilobular variety. Microscopically each lobule is found to be surrounded by a fine network of fibrous tissue, and there may also be some infiltration by the fibrous tissue between the cells at the periphery of each lobule. Much stress has been laid upon the occurrence of many proliferated bile ducts in the fibrous tissue of the unilobular cirrhotic liver; they occur in abundance; but they are not distinctive, for they are also to be seen in the multilobular cirrhotic liver, and also in the pericellular fibrosis of congenital syphilis. The spleen is often much enlarged at the same time.

A physical examination determines the presence of considerable abdominal distension from enlargement of the liver and spleen. The liver is greatly enlarged, often reaching down to the level of the umbilicus, and sometimes as far down as the right iliac fossa. The surface is smooth, hard, and often tender. The edge is hard, sharp, and well defined. Ascites is absent, or very slight in amount. The onset is gradual and insidious. Jaundice or a general increase in the size of the abdomen, with a feeling of weight in the right hypochondrium, may be the first symptoms noticed. If the jaundice becomes intense the case usually proves fatal. Evening pyrexia is present as a rule. The patient either recovers so quickly that the diagnosis is obscure, until ascites and death occur several years later; or else death occurs from cholæmia as the jaundice deepens.

Hanot's corrhosis is a familial unilobular fibrosis of the liver not due to alcohol but to some other cause not yet determined. The liver is considerably enlarged. Its surface is smooth and hard, and the edge sharp and well defined. Microscopically the fibrous tissue has a unilobular distribution, and in it plexuses of bile ducts form a prominent feature, so that it may be described as a biliary cirrhosis. The spleen is enlarged, but less so than is the liver, and it is much smaller than it is in splenomegalic cirrhosis.

The malady occurs most frequently in young people, and several members of the same family may be affected. It is not attributable to abuse of alcohol, and it is not due to malaria nor to syphilis. Jaundice, sometimes intense, sometimes slighter and chronic, is one of the commonest and earliest symptoms. There is a liability to various hæmorrhages, but not to hæmatemesis in particular. The skin may become remarkably pigmented, and there may be pyrexia. Ascites is exceptional.

#### Hæmochromatosis or Bronzed Diabetes.

In this variety of cirrhosis the liver is moderately enlarged. The spleen progressively increases in size, but it does not attain to such enormous dimensions as it does in splenomegalic cirrhosis. The splenomegaly and the cirrhosis of the liver go hand in hand. The enlargement of these organs is associated with diabetes mellitus, perhaps owing to coincident sclerosis of the pancreas. Deep pigmentation of the skin is a marked feature of the case, the bronzing being due to the deposition of an iron containing pigment which seems to result from a toxæmia giving rise to hæmolysis. The malady is rapidly fatal, but its precise pathology is not yet clear.

#### Splenomegalic Cirrhosis.

The spleen becomes enormously enlarged and the liver may be moderately increased in size. It seems not improbable that the conditions described separately in literature as primary splenomegaly, splenic anæmia, splenomegalic cirrhosis of the liver, and Banti's disease are phases of the same malady. Whether this is true or not there is no doubt that there is a group of cases in which, though the patients are young, or even juvenile, the liver turns out to be obviously cirrhotic at the post-mortem examination, while during life the main symptoms are enormous enlargement of the spleen without leucocytosis, moderate enlargement of the liver, pigmentation of the skin, stunted growth, and possibly clubbing of the fingers and hæmatemesis.

